# Editorial: Genetic adaption and metabolic response of aquatic animals to diverse water environment parameters

**DOI:** 10.3389/fphys.2022.1092413

**Published:** 2022-11-18

**Authors:** Jianlong Ge, Yu-Hung Lin, Cristian Araneda, Qingchao Wang

**Affiliations:** ^1^ Key Laboratory of Sustainable and Development of Marine Fisheries, Ministry of Agriculture and Rural Affairs, Yellow Sea Fisheries Research Institute, Chinese Academy of Fishery Sciences, Qingdao, China; ^2^ Department of Aquaculture, National Pingtung University of Science and Technology, Pingtung, Taiwan; ^3^ Departamento de Producción Animal, Facultad de Ciencias Agronómicas, Universidad de Chile, Santiago, Chile; ^4^ Department of Aquatic Animal Medicine, College of Fisheries, Huazhong Agricultural University, Wuhan, China

**Keywords:** aquatic animals, genetic adaptation, metabolic response, water parameters, temperature stress, hypoxia

Aquatic animals are continuously exposed to complex water environmental conditions including dynamic temperature range, low dissolved oxygen levels, high ammonia levels, varying salinity levels, enriched pathogenic bacteria and unbalanced food resources. Promising research has revealed that aquatic animals evolved to adapt to the extremely changeable aquatic environments for survival. The Research Topic “Genetic adaption and metabolic response of aquatic animals to diverse water environment parameters” has been conceived to set out such knowledge. Here, we offer an overview of the contents of this Research Topic, which collects 10 original research articles, one brief research report and one data report article.

Water temperature plays an essential role in the growth, survival, and reproduction of aquatic animals, and wide temperature fluctuations often put aquatic animals under either cold stress or heat stress. Due to the specific characteristics, zebrafish has been widely applied as an experimental model. In this Research Topic, cold stress and heat stress were both applied in zebrafish both *in vivo* and *in vitro*. Wang et al. indicated a close connection between oxidative stress and the cold tolerance ability of zebrafish using ZF4 cells, which could be helpful for better understanding the cold tolerance mechanism of fish. The study by Aguilar et al. revealed the explicit redistribution of energy stores and protein catabolism in zebrafish during heat stress *via* metabolomics profiling. Another study by Chang et al. was conducted on sea cucumber *Apostichopus japonicas*, which constructed a miRNA-mRNA regulatory network in the body wall of sea cucumber during heat stress.

Dissolved oxygen and ammonia are also key physical and chemical factors in aquatic ecological environment. Hypoxia stress has always been a thorny problem in aquaculture, and here authors evaluated the influence of hypoxia stress on both rainbow trout (*in vivo*) and ZFL cells (*in vitro*). Han et al. showed that triploid rainbow trout are in a defensive state under hypoxic stress caused by actual production operations (i.e., catching, gathering, transferring, or weighting). Using ZFL cells, Hu et al. also demonstrated the iron loss in cytoplasm and mitochondria during hypoxia, which leads to mitochondrial damage and ultimately cell death. Besides dissolved oxygen, ammonia has also been recognized one of the major limiting factors in intensive aquaculture systems, and aquatic animals have evolved specific ammonia detoxification strategies to cope with environmental ammonia. Wang et al. identified the differentially expressed neuropeptides in the eyestalk and cerebral ganglia of swimming crab (*Portunus trituberculatus*) under ammonia exposure, which provides a fundamental support for unraveling the regulatory roles of the neuropeptides in ammonia toxification process.

Additionally, salinity is one of the main physical properties that govern the distribution of fishes across aquatic habitats. Recently, salinization of freshwater has been one of the main causes of biological degradation of global river ecosystems. Harshini et al. reported the transcriptomic responses of kidney in *Labeo rohita* (rohu) during salinization stress, which helps to the understanding of osmoregulatory process in *L. rohita* during adaptation to salinity changes. Ranasinghe et al. identified the key regulatory role of SREBP-1 in cholesterol accumulation in livers of *Oryzias dancena* during acclimatization to fresh water and seawater.

Besides the water physicochemical factors, the water-borne pathogenic bacteria and feed resources also pose great risks to aquatic organisms. Especially, the phenotypic and genetic complexity of pathogenic bacteria significantly increase the challenges of aquatic organisms. Kang et al. reported the interspecific genetic and antibiotic resistance diversity of 192 isolates of *Vibrio Harveyi* clade collected from 2000 to 2020 from China coastal areas. In another study, Yu et al. pointed out the potential risk for the storage and transmission of resistance genes existing in antimicrobial susceptibility isolates among 33 *Vibrio scophthalmi* isolates collected from diseased marine fish intestines between 2002 and 2020. Additionally, nutrition also significantly affected the metabolism and health of fish. Cao et al. indicated that dietary hydroxyproline supplementation significantly enhanced growth performance, collagen synthesis and muscle quality of juvenile *Carassius auratus* Triploid. Wang et al. also evaluated the programming of antioxidant capacity, immunity, and lipid metabolism in *Misgurnus anguillicaudatus* larvae linked to sodium chloride and hydrogen peroxide pre-treatment during egg hatching.

In summary, this Research Topic delivers new ideas for future research in revealing genetic adaption and metabolic response of aquatic animals to diverse water environmental parameters including temperature, oxygen, ammonia, salinity, bacteria and nutrition. All the research articles in this Research Topic show that aquatic animals have evolved multiple adaptive mechanisms to deal with diverse aquatic environmental conditions. Both genetic adaptation and metabolic responses are involved in these processes, on which researchers are working from different viewpoints. We thank all the authors for their contributions and hope that this Research Topic will encourage more scientists to deepen knowledge about the relationship between aquatic animal behavior and diverse water environmental parameters.

